# A Short Manual for Monthly Nurses

**Published:** 1884-03

**Authors:** 


*A Short Manual for Monthly Nurses.* By C. J. CULLINGWORTH, M.D. Pp. 79. London: J. AND A. CHURCHILL.

This is another small book, but it is on a small subject, or at least it appeals to readers whose intellects are supposed to be limited. Nevertheless it is a good little work, well put together, clearly written, and, what is a frequent failing in works of this sort, quite free of the theoretical and the purely scientific side of obstetrics. There is here nearly everything that a monthly nurse need know; and, what is almost of equal importance, nothing that she ought not to know.

